# Use of endoscopic hand‐suturing to treat refractory bleeding from a gastric ulcer in a patient with a left ventricular assist device

**DOI:** 10.1002/deo2.369

**Published:** 2024-04-17

**Authors:** Masahiro Kondo, Tomohiro Nagasue, Takehiro Torisu, Satoshi Miyazono, Yuichi Matsuno, Takahisa Nagahata, Toru Hashimoto, Takeo Fujino, Akira Shiose, Takanari Kitazono

**Affiliations:** ^1^ Department of Medicine and Clinical Science Graduate School of Medical Sciences Kyushu University Fukuoka Japan; ^2^ Department of Cardiovascular Medicine Graduate School of Medical Sciences Kyushu University Fukuoka Japan; ^3^ Department of Advanced Cardiopulmonary Failure Graduate School of Medical Sciences Kyushu University Fukuoka Japan; ^4^ Department of Cardiovascular Surgery Graduate School of Medical Sciences Kyushu University Fukuoka Japan

**Keywords:** endoscopic hand‐suturing, endoscopic hemostasis, gastric ulcer, left ventricular assist device, ulcer bleeding

## Abstract

We herein describe a 49‐year‐old man with severe heart failure due to fulminant myocarditis who underwent left ventricular assist device implantation and received clopidogrel and warfarin as antithrombotic agents. The patient developed anemia secondary to chronic bleeding gastric hyperplastic polyps, necessitating endoscopic mucosal resection. Despite attempts to manage post‐endoscopic mucosal resection bleeding from a gastric ulcer by endoscopic hemostasis using hemostatic forceps, local hemostatic agents, and polyglycolic acid sheets, the bleeding persisted. Hemostasis of the refractory bleeding was finally achieved by endoscopic hand‐suturing of the ulcer. One month later, the ulcer was almost completely scarred. This case has important clinical value in that it demonstrates the efficacy of endoscopic hand‐suturing even in challenging cases such as refractory bleeding gastric ulcers in patients with left ventricular assist devices.

## INTRODUCTION

Left ventricular assist device (LVAD) implantation is an essential treatment modality for severe heart failure. However, a potential complication that can be difficult to control is gastrointestinal bleeding. Such bleeding may occur in patients with LVADs for a variety of reasons, including anticoagulation therapy, changes in von Willebrand factor, and decreased circulatory activity.^1^ We frequently encounter cases in which gastrointestinal bleeding is difficult to control.

Endoscopic hand‐suturing (EHS) is an endoscopic continuous suturing technique involving the use of an absorbable suture with spines and a needle holder that passes through a scope, allowing continuous linear suturing of the mucosal layer.^2^ We herein report a case of a post‐endoscopic mucosal resection (EMR) refractory bleeding gastric ulcer that was successfully treated with EHS in a patient with an LVAD.

## CASE REPORT

**FIGURE 1:**
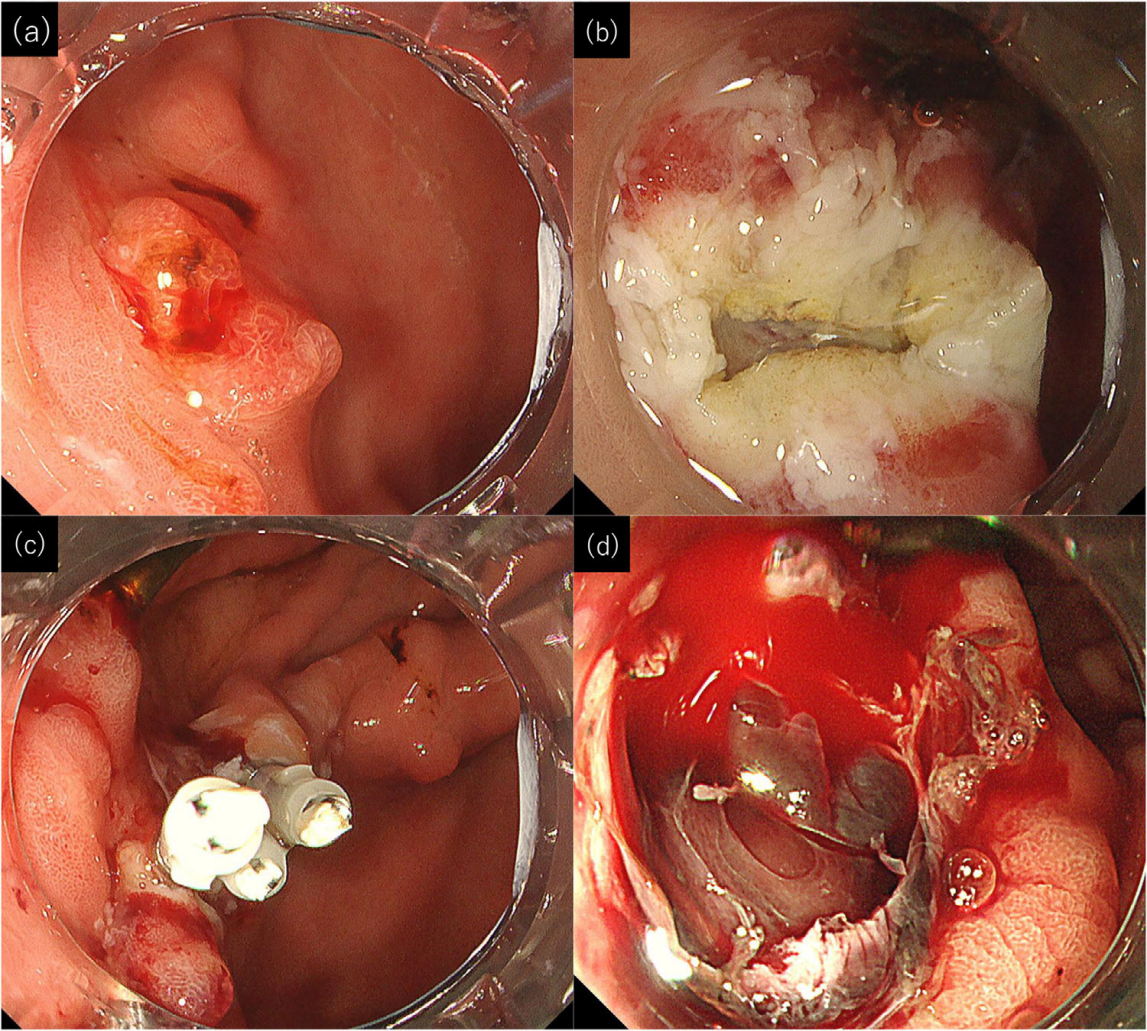
Endoscopic findings during endoscopic mucosal resection (EMR) and bleeding of lesions. (a) Endoscopy immediately prior to EMR. An erythematous polyp with hemorrhage was seen on the anterior wall of the antrum. (b) Endoscopy after EMR. No bleeding from the ulcer was observed. (c) Endoscopy after EMR. The post‐EMR ulcer was closed by clips. (d) Endoscopy 7 days after EMR. Pulsatile bleeding from the post‐EMR ulcer was observed. Clots were observed at the base of the ulcer.

**FIGURE 2:**
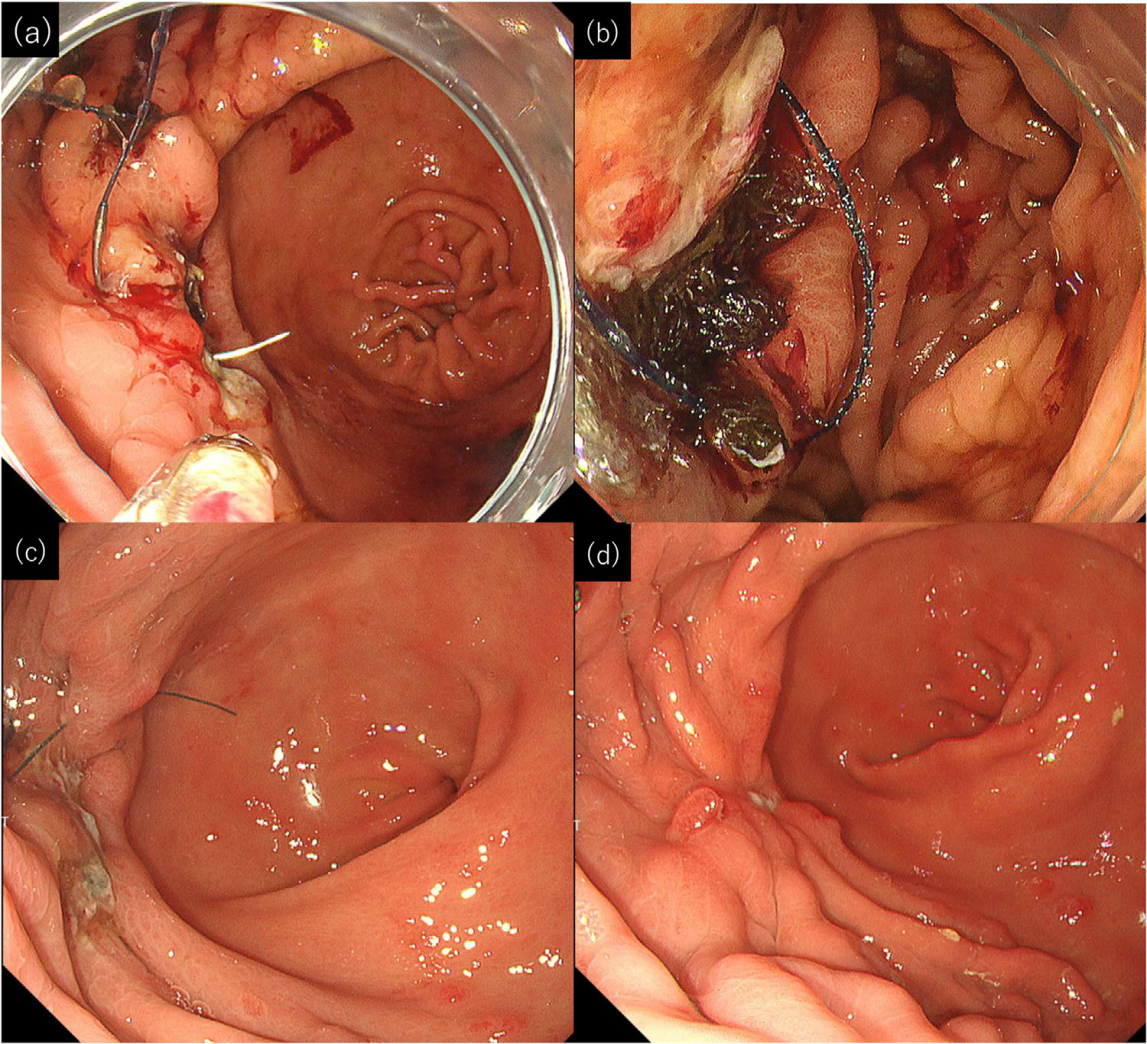
Endoscopic hand‐suturing for treatment of endoscopic mucosal resection (post‐endoscopic mucosal resection) ulcer. (a) Suture placement began at the edge of the ulcer, and a linear continuous suture was performed. (b) polyglycolic acid sheets, reddish‐black with blood, were filled and tightened under the sutures. (c) Endoscopy the day after endoscopic hand‐suturing. The ulcer remained closed. (d) Endoscopy 25 days after endoscopic hand‐suturing. The ulcer had largely scarred, leaving a slight mucosal defect.

A 49‐year‐old man presented to our institute for treatment of anemia secondary to chronic bleeding from gastric hyperplastic polyps. He had severe heart failure due to fulminant myocarditis and had undergone implantation of an LVAD (HVAD System; Medtronic), and he was receiving clopidogrel and warfarin as antithrombotic agents and vonoprazan as an acid secretion inhibitor. Esophagogastroduodenoscopy (EGD) showed erythematous polyps from the body to the antrum of the stomach; they were suspected to be hyperplastic polyps (Figure 1a). Three polyps in the body and antrum regions were removed by EMR (Figure 1b), and the wound was closed with clips because of the high risk of post‐EMR ulcer bleeding (Figure 1c). Postoperatively, the patient was treated with vonoprazan and followed a fasting regimen. However, he developed melena on postoperative day (POD) 3. Subsequent EGD revealed bleeding from a post‐EMR ulcer in the antrum, which was treated with radiofrequency coagulation (Figure 1d). The patient discontinued warfarin. Melena was observed on PODs 5, 7, 8, 9, 10, and 11. We attempted to manage the bleeding each time with radiofrequency coagulation, application of polyglycolic acid (PGA) sheets, and local hemostatic agents. However, these conventional hemostatic methods were unsuccessful. The patient's anemia progressed, necessitating frequent blood transfusions. On POD 13, EGD again showed active bleeding from an ulcer in the antrum. We performed radiofrequency coagulation to stop the bleeding, and we attempted to close the ulcer with EHS. The EHS procedure was performed using a V‐Loc 180 absorbable barbed suture (VLOCL0604; Covidien) and a flexible needle holder (Olympus Co., Ltd.; Figure 2a). New PGA sheets were placed under the suture, and fibrinogen with thrombin was applied before the suture was tightened (Figure 2b). The ulcer was completely closed with four stitches, and the procedure took 70 min. The day after treatment, EGD showed that the wound was closed and was not bleeding. The patient's clinical course was smooth thereafter, and the bleeding did not recur. He started eating on POD 19 and resumed warfarin on POD 24. At 25 days post‐EHS, the ulcer showed scarring with only a slight mucosal defect remaining (Figure 2c,d).

## DISCUSSION

In this case, EMR was performed to remove gastric hyperplastic polyps that had caused anemia in a patient who had severe heart failure and had undergone LVAD implantation. The ulcer bled after EMR, and repeated bleeding occurred despite multiple appropriate endoscopic treatments using hemostatic forceps, local hemostatic agents, and PGA sheets. Because EHS resembles surgical suturing using a needle and thread, it is expected to provide more reliable and durable wound closure than conventional endoscopic closure techniques using clips or indwelling snares. Especially in the stomach, ulcers are protected from various stimuli such as gastric acid, and this protection is considered to greatly reduce the risk of rebleeding. A PGA sheet is a tissue reinforcement material often used in gastrointestinal surgery to reinforce sutures and prevent air leakage. The surrounding tissue infiltrates between the PGA fibers, and biological tissue is regenerated in the process of degradation by biological reactions. The main issue in the application of PGA sheets is the persistence of local fixation. In the present case, it was possible to firmly fix the PGA sheets locally by placing them under the suture and then tightening the suture. We consider that this technique further enhanced the hemostatic effect in this case. Several reports have examined the feasibility and efficacy of EHS for mucosal defects after endoscopic submucosal dissection^3^, ^4^; however, the patients in these reports were treated with EHS immediately after treatment, and the modifying effects of the endoscopic procedure, such as fibrosis, were thought to be minimal. In the present case, multiple radiofrequency coagulations were performed before EHS, and we expected that the procedure itself would be difficult to perform with conventional endoscopic closure because of weakness, edema, and fibrosis of the tissue. In fact, EHS was sufficient to close the ulcer, and the wound remained closed as shown by EGD the day after EHS.

LVAD implantation has been on the rise in recent years as a definitive treatment for end‐stage heart failure and a bridge to heart transplantation.^5^ Gastrointestinal bleeding is one of the most common adverse events in patients with LVADs, affecting 21% to 36% of patients.^6^, ^7^ The causes are multiple, including combination antiplatelet therapy and vitamin K antagonist therapy, activation of fibrinolytic pathways, acquired von Willebrand factor deficiency, and a tendency toward angiodysplasia; bleeding may also be influenced by the poor general condition of patients requiring such devices.^1^ Research has shown that patients with LVADs who secondarily develop gastrointestinal bleeding often experience recurrent bleeding and require repeated therapeutic interventions.^8^ Although primary hemostasis was successfully achieved with each endoscopic hemostatic procedure in this case, repeated high‐frequency coagulation may have led to prolonged healing of the ulcer and a vicious cycle of recurrent bleeding due to the patient's predisposition.

Although EHS has many advantages, including reliable and durable wound closure independent of wound size, it remains a limited procedure. A certain level of endoscopic skill is required for successful EHS. The specific skills required are not certain, but it is likely that successful EHS requires a skill level comparable to that of endoscopic submucosal dissection. It is also important to become familiar with EHS through prior in vitro training. The number of facilities in which EHS can be performed is also limited; thus, it is expected that the technique will be disseminated through further accumulation of cases.

In conclusion, a patient who had undergone LVAD implantation for severe heart failure developed a refractory bleeding gastric ulcer after EMR. The ulcer was successfully treated with EHS with no rebleeding.

## CONFLICT OF INTEREST STATEMENT

None.
